# Hypertrophied cruciate ligament in high performance weightlifters observed in magnetic resonance imaging

**DOI:** 10.1007/s00264-012-1528-3

**Published:** 2012-03-25

**Authors:** Piotr Grzelak, Michał Podgorski, Ludomir Stefanczyk, Marek Krochmalski, Marcin Domzalski

**Affiliations:** 1 Department of Radiology, Medical University of Lodz, 22 Kopcinskiego Street, 90-153 Lodz, Poland; 2 Department of Orthopaedics, Medical University of Lodz, Lodz, Poland; 3 Medical Magnus Clinic, Lodz, Poland

## Abstract

**Purpose:**

In a group of high performance weightlifters increased values of the cruciate ligaments (CLs) cross-sectional areas were observed. The purpose of this research was to investigate if repeated heavy workouts increase the volume of those structures.

**Methods:**

The knee examinations were performed with an 1,5T MRI system. The area of the anterior cruciate ligament (ACL) and the posterior cruciate ligament (PCL) midsubstance cross-section were evaluated in T1-weighted images with administration of contrast medium in a group of nine athletes. A control group of 19 participants was also examined using the same protocol.

**Results:**

Significant differences of the ACL and the PCL midsubstance cross-sectional areas were observed between groups. The area of the CLs' midsubstance and the onset of training were strongly negatively correlated and the PCL cross-sectional area was strongly positively correlated with the duration of training.

**Conclusion:**

This research is the first description of the CLs hypertrophy, which is probably caused by heavy training that was started about the age of puberty. The age of training onset seems to have a greater impact on the hypertrophy process than the training duration. Knowledge of the phenomenon of cruciate ligament overgrowth is vital for orthopaedics because, possible changes of the CLs mechanical properties and three-dimensional orientation, may affect the incidence of trauma and reconstruction procedures technique.

## Introduction

Cruciate ligaments (CL) are intra-articular but extrasynovial structures stabilising knee joint movements. The anterior cruciate ligament mainly prevents anterior tibial translation and, to a lesser extent, internal tibial rotation. The posterior cruciate ligament prevents the femur from sliding forward on the tibia and it stabilises the knee in a rotational fashion. The midsubstance of CLs has been described exhaustively in the literature as far as the normal anatomy and the histology are concerned [1, 2]. Much attention has also been devoted to CL insertions when it comes to the tunnel placement in CL reconstruction techniques [3–5]. The morphology of the midsubstance of CLs, in terms of their hypertrophy, is a much less researched area.

The weightlifters are a very specific type of athlete and most literature concerns spine and muscle problems in this population [6–8]. It is well known that repeated heavy workouts increase the volume of many soft tissue structures such as muscles, tendons and extra-articular ligaments. This problem, however, has not been investigated in the knee joint. Possible enlargement of CLs may change the three-dimensional orientation of the ligaments and cause impingement. Therefore awareness of such a phenomenon is very important in diagnosing knee problems and planning the treatment in this group of patients. To the best knowledge of the authors, we present the first magnetic resonance imaging (MRI) study analysing the volume of the cruciate ligaments among high-performance weightlifters.

## Materials and methods

**Fig. 1:**
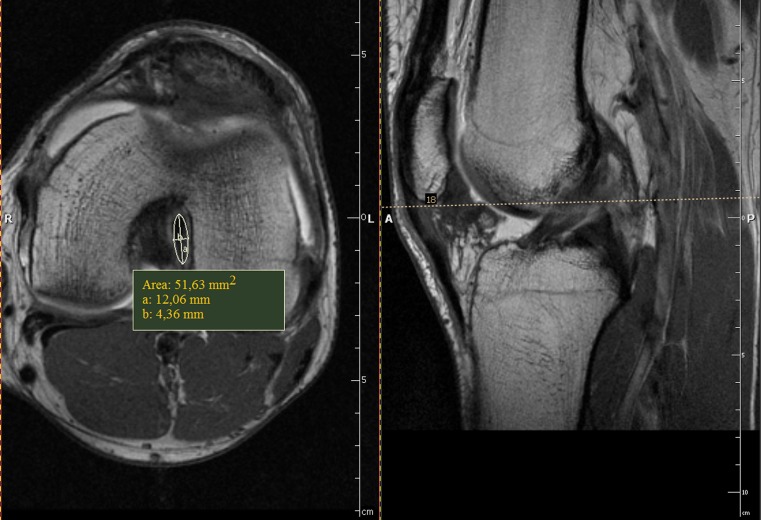
T1-weighted MR images with intra-articular administration of contrast medium. *Left side*: measurement of the anterior cruciate ligament (ACL) surface area using a modifiable ellipsoid on the axial section. *Right side*: measurements level (*dashed line*) on sagittal section

**Fig. 2:**
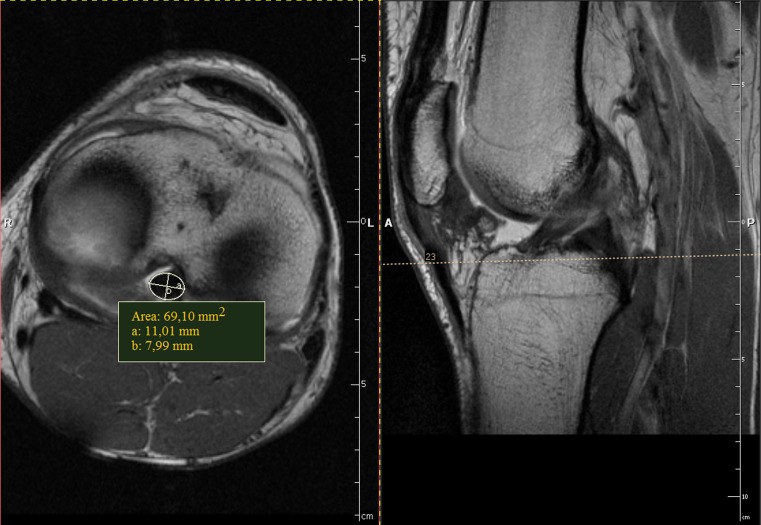
T1-weighted MR images with intraarticular administration of contrast medium. *Left side*: measurement of posterior cruciate ligament (PCL) surface area using a modifiable ellipsoid on axial section. *Right side*: measurements level (*dashed line*) on sagittal section

The database at our radiology department was searched for MRI data of knees in high-performance weightlifters. Data about the age, weight and height was obtained. From this group we selected nine athletes with no history of knee trauma whose MRI results were negative for cruciate ligament problems. The main indications for MRI in this group were patellar problems and minor meniscal lesions. None of the weightlifters had subsequent knee surgery. Seven of the participants had both of their knees evaluated and in two weightlifters only one knee was available for assessment. Information about the time span of training participation and the age of training onset was collected from the study group. The control group consisted of 19 age-matched males, with no history of knee trauma and negative MRI findings. There were no statistically significant differences in height and weight between the control and study groups. Participant data for both groups are presented in Table 1. The same imaging protocol was used for both groups. The examinations were performed with an Avanto 1,5T MRI system (Siemens, Germany), using the dedicated coil. All images were analysed retrospectively on a work station (Exchibeon, Pixel Technology, Poland) using software that allowed for the measurement of surface area CL by using a modifiable ellipsoid (Figs. 1, 2). The dimensions (cross-section) of the CL midsubstance were evaluated on the basis of MRI images. For the statistical analysis, we used the T1-weighted images with administration of contrast medium (Artirem, Guerbet, Germany) into the joint for better separation of Cls. The measurement of the ACL was performed at half the length of the ligament. Level of measurement was determined on the basis of the sagittal section in the middle distance from the femoral attachment to the tibial attachment of the ligament. PCL measurement was carried out in the bottom third of the length of the ligament. Level of measurement was determined based on a line parallel to the plane of the intercondylar eminence on the sagittal section. The morphologically corrected picture and the typical signal of the CLs were taken as evidence of absent CL injury. In statistical analysis the Manny-Whitney test, the t-test and the Spearman's rank correlation test were employed. P < 0.05 was taken to be significant.

**Table 1:** Participant and control group participant data

Characteristic	Weightlifters (*N* = 9)				Control (*N* = 19)			
	Mean	Minimum	Maximum	Standard deviation	Mean	Minimum	Maximum	Standard deviation
Age (years)	26.1	21	34	4.2	26.6	19	36	5.3
Weight (kg)	92.6	78	120	14.8	86.6	72	106	8.8
Height (cm)	177.6	168	188	6.4	179.8	169	190	5.4
BMI	29.1	27.1	34.7	2.7	26.8	23.8	31.7	2.2
Training participation (years)	15.5	10	25	4.7				
Age of training onset (years)	10.6	9	12	0.8				

*N* number of participants in the group, *BMI* body mass index

## Results

The area of the ACL and the PCL midsubstance cross-sections for the study and for the control groups are shown in Table 2.

**Table 2:** The area of ACL and PCL midsubstance cross-sections

Group	ACL		PCL	
	Mean (mm^2^)	Range (mm^2^)	Mean (mm^2^)	Range (mm^2^)
Weightlifters (*N* = 16)	71.7	52.9–111.2	64.48	52–88.1
Control (*N* = 19)	40.56	23.83–59.1	44.98	31.3–71

*N* number of participants in the group, *ACL* anterior cruciate ligament, *PCL* posterior cruciate ligament

**Fig. 3:**
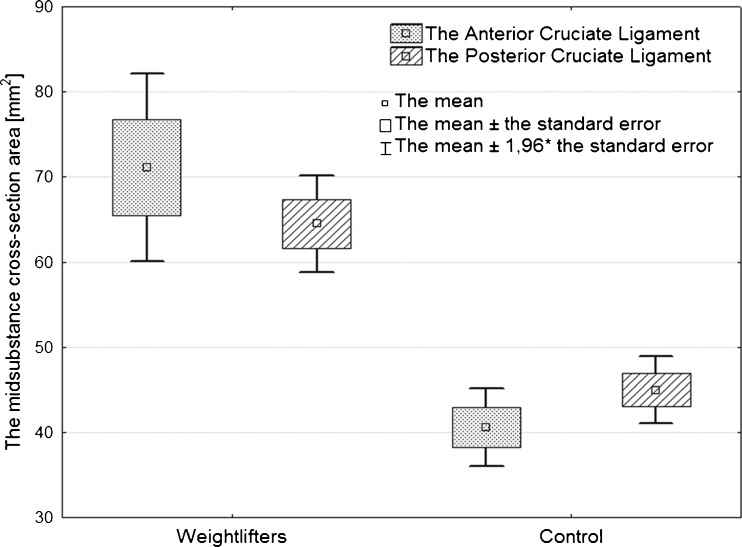
Box-and-whiskers diagram displaying differences of cruciate ligaments (CLs) midsubstance cross-sectional areas between weightlifters and control

The Manny-Whitney test confirmed that the cross-sectional areas of the ACL and PCL were significantly higher in weightlifters than in controls (Fig. 3). The Spearman's rank correlation test indicated strong negative correlation between the area of the CL midsubstance and the onset of training with a coefficient of −0.56 for the ACL and −0.71 for the PCL. Using the same test we also found a strong positive correlation (0.6) between the years of training participation and the area of the cross-sections of the PCL.

## Discussion

MRI is a widely used imaging modality providing information about the natural structure of the CLs [9]. Anatomical studies have revealed that the tibial and femoral insertions of the ACL in comparison to the ligament midsubstance are approximately 3.5 times larger, and the insertions of the PCL are also over three times larger [3]. When the synovial membrane was dissected before measuring the femoral insertion of the ACL, the ratio was 2.5 [4]. Cadaveric studies have shown that the cross-sectional shape of ACL midsubstance changes with the angle of knee flexion, but is generally larger in the anterior–posterior direction. Furthermore, whereas the cross-sectional shape of the ACL is rather irregular, its cross-sectional area is rather constant and it increases from the femur to the tibia, as follows: 34 mm^2^ proximally, 33 mm^2^ mid-proximally, 35 mm^2^ at mid-substance level, 38 mm^2^ mid-distally, and 42 mm^2^ distally [2]. Values of the area in the distal and mid-distal parts are similar to those presented in the control group confirming the utility of MRI in evaluation of the CL's cross-sectional area.

To the authors' knowledge, the process of CL hypertrophy has not been described in the literature. Changes in CL morphology are observed in patients suffering from CL injury. Studies have looked at CL elongation and diameter reduction manifesting as laxity in the knee joint [10]. The study group comprised of high level athletes with no history of CL trauma which allows us to hypothesise that the factors responsible for the hypertrophy process may be:

1. Early onset of training. Puberty is the time of extensive growth. All athletes in our study began training before or during their pubertal growth spurt. Tissues prone to growth in that period could undergo hypertrophy in response to extensive training. Moreover, youths demonstrate higher potential for regeneration processes, which may also be manifested by intensive cell proliferation due to strain. An investigation of high school basketball players found that the ACL midsubstance had a cross-sectional area in the insertion point of 36.1 and 48.9 mm^2^ for females and males, respectively [11]. Those participants were not professional players and no control group was analysed, but those results may indicate that the process of CL hypertrophy is increased in younger people, in response to training.
2. Increased blood supply. The middle genicular artery (MGA) is the main source of blood for the CLs. The foetal CLs have been shown to resemble the adult knee in almost every way except for vascularity [12]. The branches of the MGA seen in foetuses are usually lost in adults [13]. There is no data about the age of artery involution so it is probable that starting training at a young age can delay this process thus providing sufficient blood supply for the process of CL hypertrophy.
3. Longitudinal CL stress. Weightlifting training comprises exercises where the athletes crouch with maximally flexed knees. Moreover, they usually increase the load to their knees by lifting additional weight. In this position the CLs are highly taut and high longitudinal strain is exerted. This may be the impetus for the proliferation of midsubstance fibroblasts [14]. Another argument confirming this hypothesis may be the presence of midsubstance regions composed of fibrocartilagenous tissue. It indicates that the fibrocartilagenous hypertrophy can be a response to exposure of this part of the tendon to a pressure [15].

Statistical analysis supports our hypothesis. Strong negative correlation between the area of the CL midsubstance and the onset of training indicate that the younger the athlete was at the moment of starting to train, the greater the hypertrophy of the CLs. The relationship between the duration of training and the area of CLs was not so clear. The age of training onset seems to be more clearly linked to the process of CL hypertrophy. The ultimate load to failure and stiffness of CLs decrease significantly with age [2]. The influence of hypertrophied CL midsubstance on those parameters might play a protective role in CL injury but further investigation is needed to confirm this hypothesis.

Our study proves that cruciate ligaments are hypertrophied in weightlifters similarly to other soft tissue structures. Currently, we cannot predict the role this may play for the athletes' knees in the future but impingement between ligaments is possible. On the other hand overgrown CLs might support the knee joint stability and have a protective role in ACL injury especially in patients with a defined risk factor such as steeper lateral tibial plateau slope [16]. Notwithstanding the foregoing we think that the knowledge of this phenomenon may be helpful for many doctors treating this specific patient population.
